# Loss of Breathing Modulation of Heart Rate Variability in Patients with Recent and Long Standing Diabetes Mellitus Type II

**DOI:** 10.1371/journal.pone.0165904

**Published:** 2016-11-01

**Authors:** Ana Leonor Rivera, Bruno Estañol, Ruben Fossion, Juan C. Toledo-Roy, José A. Callejas-Rojas, José A. Gien-López, Guillermo R. Delgado-García, Alejandro Frank

**Affiliations:** 1 Instituto de Ciencias Nucleares, Universidad Nacional Autónoma de México, México City, México; 2 Centro de Ciencias de la Complejidad, Universidad Nacional Autónoma de México, México City, México; 3 Laboratorio de Neurofisiología, Departamento de Neurología y Psiquiatría, Instituto Nacional de Ciencias Médicas y Nutrición Salvador Zubirán, México City, México; 4 Departamento de Medicina Interna, Hospital Universitario "Dr. José Eleuterio González", Universidad Autónoma de Nuevo León, Monterrey, Nuevo León, México; 5 Colegio Nacional, México City, México; University of Washington, UNITED STATES

## Abstract

Healthy subjects under rhythmic breathing have heart interbeat intervals with a respiratory band in the frequency domain that can be an index of vagal activity. Diabetes Mellitus Type II (DM) affects the autonomic nervous system of patients, thus it can be expected changes on the vagal activity. Here, the influence of DM on the breathing modulation of the heart rate is evaluated by analyzing in the frequency domain heart interbeat interval (IBI) records obtained from 30 recently diagnosed, 15 long standing DM patients, and 30 control subjects during standardized clinical tests of controlled breathing at 0.1 Hz, supine rest and standing upright. Fourier spectral analysis of IBI records quantifies heart rate variability in different regions: low-frequencies (LF, 0.04–0.15 Hz), high-frequencies (HF, 0.15–0.4 Hz), and a controlled breathing peak (RP, centered around 0.1 Hz). Two new parameters are introduced: the frequency radius r_f_ (square root of the sum of LF and HF squared) and β (power of RP divided by the sum of LF and HF). As diabetes evolves, the controlled breathing peak loses power and shifts to smaller frequencies, indicating that heart rate modulation is slower in diabetic patients than in controls. In contrast to the traditional parameters LF, HF and LF/HF, which do not show significant differences between the three populations in neither of the clinical tests, the new parameters r_f_ and β, distinguish between control and diabetic subjects in the case of controlled breathing. Sympathetic activity that is driven by the baroreceptor reflex associated with the 0.1 Hz breathing modulations is affected in DM patients. Diabetes produces not only a rigid heartbeat with less autonomic induced variability (r_f_ diminishes), but also alters the coupling between breathing and heart rate (reduced β), due to a progressive decline of vagal and sympathetic activity.

## Introduction

The autonomic nervous system modulates the cardiac cycle through central (e.g. vasomotor and respiratory centers) and peripheral (e.g. arterial pressure and respiratory movements) oscillators that manifest as short and long-term oscillations in the heart rate. Parasympathetic modulation decreases the heart rate and cardiac contractility, whereas activity of the sympathetic branch opposes these effects and regulates peripheral vasoconstriction [1]. Loss of this balance, which is common in Diabetes Mellitus type II (DM) patients, is associated with a high risk of cardiovascular disease due to autonomic neuropathy [2–6]. Vagal and sympathetic modulation of the sinus node can be evaluated through heart rate variability (HRV), which provides a noninvasive method for understanding cardiac autonomic control [6–9].

A common measure of HRV is the power spectral density (PSD) of the signal, which reflects how the variance of the IBI time series is distributed as a function of frequency (*f*), revealing the cyclic components responsible for variability in the recording period [10]. PSD is defined as the square of the Fourier Transform of the interbeat interval (IBI) signal. Commonly, different spectral bands are distinguished in the PSD: a very low frequency region (VLF, *f*<0.04 Hz), a low frequency region (LF, 0.04<*f*<0.15 Hz), and a high frequency region (HF, 0.15<*f*<0.4 Hz) [9–12]. VLF obtained from short-term recordings (less than 5 minutes) is not a reliable measure because it is sensitive to the trend of the baseline and/or the trend removal algorithm employed in the analysis [7]; therefore, VLF is not considered in this work. HF is generally associated with cardiac vagal activity [9–12] while LF is considered as a marker of sympathetic modulation [9] or as a parameter related to the baroreceptor control which is mediated by both sympathetic and vagal influences [11, 13, 14]. The LF/HF ratio has been used as a measure of the sympathetic/vagal balance, an increase of LF/HF is assumed to reflect a shift towards sympathetic dominance, whereas a decreases of this index corresponds to a parasympathetic dominance [7, 11–14]. However, one has to be cautious with this interpretation of the LF/HF ratio [15] because it is confused in patients with acute myocardial ischemia, exercise [16] or heart failure [17–18].

Analysis of short-term and/or long-term HRV has been used in the past to study DM [3, 19–26]. Long-term records show reduced power in all spectral bands [22–27], failure to increase LF during standing related to a depressed baroreceptor sensitivity [24, 25], a leftward shift of the central frequency of the LF band towards lower frequencies [25] without a significant difference in the LF/HF ratio for diabetic patients compared to control subjects [24]. For short-term HRV, changes in the time—domain parameters, especially in variance, skewness and kurtosis, can be used as early warnings of DM autonomic neuropathy [27]. However, there are no conclusive studies of short-term records in the frequency-domain.

In the frequency domain, the PSD of HRV of healthy subjects exhibits a respiratory band that is thought to be mediated by fluctuations of vagal-cardiac nerve traffic, so it can be an index of vagal activity [8]. Respiratory-frequency rhythms in autonomic nerves are translated into changes in discharge frequency of the cardiac sinoatrial node; due to the low-pass-filter characteristics of the autonomic-cardiac innervations, the width of the respiratory band of the PSD depends on the breathing rate and is wider for slow than for fast breathing [28, 29]. At usual breathing frequencies, autonomic sensory inputs are more likely to influence sympathetic and vagal firing during expiration than during inspiration, which produces a modulation of the heart rhythm [30].

The objective of the present research is to evaluate the influence of a disease such as DM on the cardiovascular response using non-invasive short-term (5-min) IBI records. These measures can be easily obtained during routine clinical examination. We investigate how cardiac dynamics is affected by DM using a frequency-domain analysis of IBI records taken during the standard cardiovascular reflex tests established by the American Diabetes Association (ADA) [4, 31]: heart rate measurement during supine rest, standing upright, and controlled breathing at 0.1 Hz. To study the impact of DM on heart rate, the breathing modulation of the heart rhythm was analyzed in the frequency domain.

## Research Design and Methods

The present study is based on non-invasive short-term measurements of blood pressure taken at each heartbeat using a Portapres^®^ device of Finapres Medical Systems, The Netherlands [23, 26, 32]. The Portapres^®^ is a portable ambulatory blood pressure monitoring tool that records multiple hemodynamic parameters, including blood pressure (systolic, diastolic, mean), heart rate and interbeat interval. It consists of a lightweight main unit worn by the subject on a waist belt and a frontend unit worn on the wrist connected to a finger cuff to perform the measurement. The device continually records the pressure waveform at the finger and automatically reconstructs the brachial artery pressure waveform, which allows to derive the other hemodynamic parameters. Time is recorded with 1 ms precision while blood pressure values are recorded with 1 mmHg precision. Previous studied comparing the Portapres^®^ to direct invasive intra-aortic measurements have shown that pressure and pulse can be determined reliably within the Association for the Advancement of Medical Instrumentation (AAMI) requirements [33].

The study population was composed by 75 subjects clinically classified in 3 distinct groups as follows:

- 30 control subjects, 13 males, 17 females, 21 to 50 years of age (with a mean and standard deviation of 39±12 yr) with body mass index (BMI) from 19.1 to 28.5 kg/m^2^ (25±3 kg/m^2^). Subjects were accepted as controls if they were healthy, did not smoke, had no cardiac diseases, were not hypertensive, had blood pressure levels of 120/80 mmHg or less, and did not take anticholinergic or adrenergic medication.
- 30 patients with recently diagnosed DM type II, 14 males, 16 females, 38 to 48 years of age (41±5 yr) with BMI from 25.8 to 29.4 kg/m^2^ (27±2 kg/m^2^). They were part of a cohort of patients with metabolic syndrome who underwent yearly an oral glucose tolerance test (GTT) of 75 g for two hours, and were declared diabetic when the GTT exceeded 200 mgs/dl. In their previous medical examination, they were considered to have metabolic syndrome but did not take medications.
- 15 patients with long standing DM type II diagnosed 15±9 years ago, 5 males, 10 females, 20 to 76 years of age (53±18 yr) with BMI from 18.1 to 36.8 kg/m^2^ (27±6 kg/m^2^). DM patients underwent a medical diagnosis by an expert, had blood glucose levels larger than 200 mg/dL at two hours in a glucose tolerance test, in agreement with the recommendations of the “Asociación Mexicana de Diabetes” and the 2011 “American Diabetes Association” [31].

Control subjects and DM patients abstained from caffeine, beta-blockers, anticholinergics, antihistamines, opioids and adrenergic medicaments during the 48 hours previous to the examination. Previous to the cardiovascular monitoring, all subject underwent a 12-lead ECG study to exclude patients with arrhythmia.

All subjects provided written informed consent, underwent a physical examination and had their medical history screened. The Ethics’ Committee of Instituto Nacional de Ciencias Médicas y Nutrición “Salvador Zubirán” from México approved the protocol for data recording.

The hemodynamic parameters of all patients were recorded during three standardized clinical tests recommended by ADA [24]: supine position, followed by standing upright, and finally a controlled breathing test at 0.1 Hz. The tests were carried out as follows. Initially, each subject relaxed on a bed for one minute, then IBI data were registered while the subject was in a supine rest position for 5 minutes. Afterwards, the subject was made to stand up, 1 min was allowed for the hemodynamic variables to adapt to the new posture and another IBI measurement was made for the following 5 minutes. Finally, a third IBI registration of 5 minutes was realized while the subject was breathing rhythmically at 0.1 Hz. In this test the subject was asked to inspire and expire rhythmically at six breathing cycles per minute, 5 seconds in and 5 seconds out. A medical assistant kept track of time using a stopwatch and provided verbal and visual (rhythmically raising and lowering a hand) instructions to the patient. A record length of 5 minutes has been established as a standard time interval for short-term heart rate variability analysis [7, 34–36].

All IBI records obtained with the Portapres^®^ were visually inspected for artifacts, ectopic beats or abnormal intervals. No ectopic beats (identified as intervals with values less than half of the mean of the series) or obvious artifacts were found in the data. Also, no initial transient effects could by identified in the signals, indicating that the 1min adaptation interval between change in posture was sufficient.

**Fig 1:**
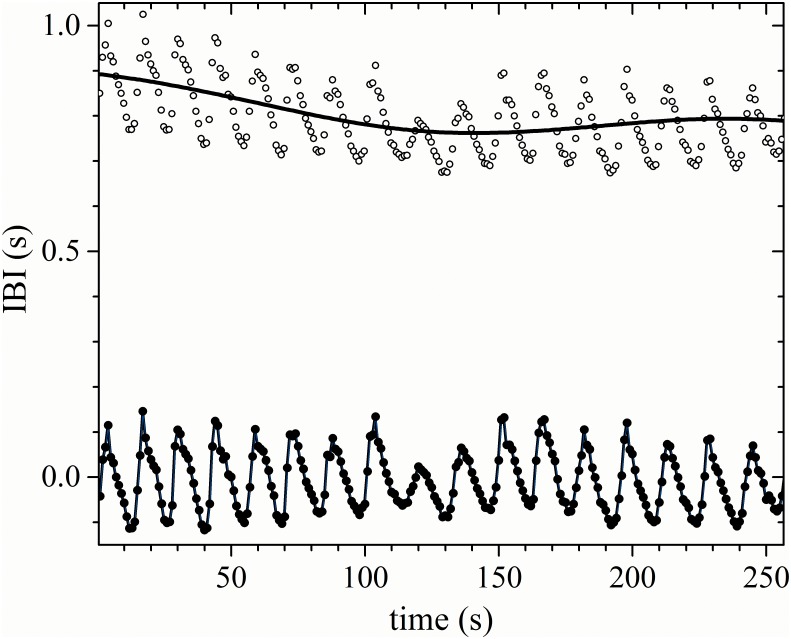
Interpolated signal. Original IBI signal (open circles), with the median superimposed (continuous curve), with the detrended (filled circles), and the regularly interpolated IBI data (line). The data corresponds to a typical recently diagnosed DM patient during a controlled breathing test.

Signals were detrended to eliminate the contributions of artificial trends to the low-frequency regions of the spectrum, and to normalize the baseline to zero for all recordings. The global trend of each time series was subtracted using the median of the time series. Subsequently, in OriginPro^®^ 9.1, 64 bits, an equally-spaced time series (with a sampling rate of one second) was generated from the irregularly sampled IBI data using a cubic spline interpolation. The equally-spaced time series differed only slightly from the original signal, but interpolation was performed to ensure the applicability of spectral methods based on the Fourier transform (see Fig 1). To perform the spectral analysis of the heart rhythm, a Fast Fourier Transform (FFT) algorithm was applied to short segments of 256 successive data points of the resulting detrended and interpolated IBI record (equivalent to the first 4 minutes and 16 seconds of the record). Then, LF, HF, and the power of the respiratory peak (RP) related to controlled breathing were evaluated.

**Fig 2:**
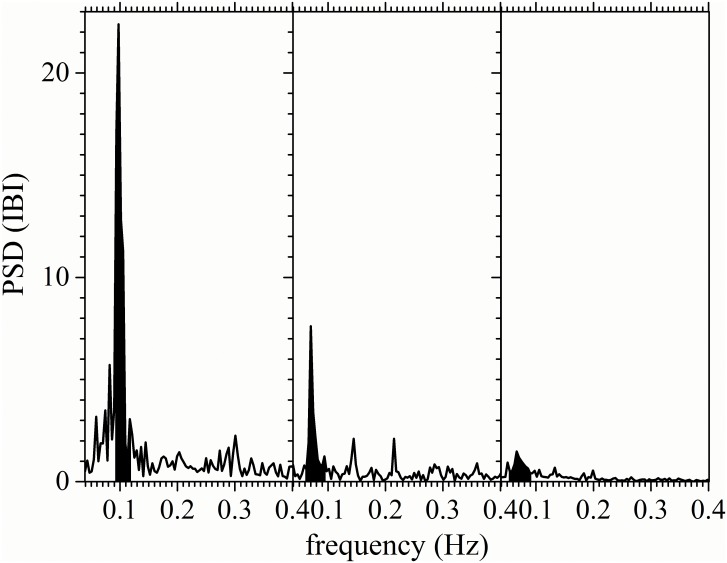
Respiratory peak on the frequency domain. Typical power spectral density (PSD) of IBI records during a controlled breathing test for a control subject (left-hand panel), a recently diagnosed DM patient (middle panel) and a long-standing DM patient (right-hand panel). The dominant respiratory peak (RP) region is shaded black.

It is known that the respiratory resonant frequency of 0.1 Hz can induce periodic modulations in the cardiac rhythm with a frequency that depends on each person around 0.1 Hz [37]. In our study, for control subjects, the respiratory modulation of the cardiac activity during a controlled breathing test is evident in the Fourier transform of the IBI signal as a sharp increase in spectral power in a narrow band around the breathing frequency of 0.1 Hz (dark shaded region in the left panel of Fig 2). We thus propose to utilize the power contained in this band as a way to quantify the strength of the cardiorespiratory coupling. We define the Respiratory Peak (RP) as the power in the frequency band centered at the peak in the 0.1 Hz region spanning from the starting rising position to the end decreasing point (generally from 0.086 Hz to 0.113 Hz). If there is no a clear respiratory peak, as in the case of long-standing diabetes mellitus patients, the power in the region from 0.086 to 0.113 Hz is evaluated as the RP.

In order to quantify the relative importance of the cardio-respiratory coupling in a way that is not sensitive to the total power in the spectrum, we propose a new parameter, β, defined as the ratio between the power of the respiratory peak RP and the combined LF plus HF power:

$$\beta =\frac{\text{RP}}{\left(\text{LF}+\text{HF}\right)}.$$

This parameter evaluates the capacity of the breathing modulation of the heart rate as a fraction of the total power in the frequency domain. Note that the β parameter is easiest to define in the case of the controlled breathing tests, because here the Fourier spectra of the IBI records usually have a dominant peak centered on the respiratory frequency (clearly associated with breathing), which might not be present during the supine rest and/or the standing upright tests.

Traditionally, LF and HF spectral indices are considerably reduced as diabetes evolves [6]. We found the same result, as seen in Table 1. LF and HF are statistically significant different between control and long-standing DM subjects, with p-values less than 0.05. The LF/HF ratio estimates the balance of sympathetic modulation (indicated by LF) and vagal activity (associated with HF) [11–14]. However, in our study this ratio does not make a significant statistical distinction of the loss of autonomic balance in diabetic patients (see Table 1). Instead, we propose a new parameter, the “frequency radius” r_f_, defined as

$${\text{r}}_{\text{f}}=\sqrt{{\text{LF}}^{2}+{\text{HF}}^{2}},$$

as an alternative way to measure the total modulation capacity of the autonomic nervous system, including contributions from both the sympathetic and the vagal branches. One way to understand this new quantity is to think of each (LF, HF) pair of each subject as the coordinates of a point in the LF—HF plane. In this representation, the LF/HF ratio is related to the slope of the line connecting that point to the origin, while the new parameter of the frequency radius r_f_ corresponds simply to the distance from the point (LF, HF) to the origin. We will demonstrate that this parameter gives a statistical significant separation of control and diabetic subjects.

**Table 1:** Characteristic parameters of the IBI spectrum during the controlled breathing test in the frequency domain.

	Peak position	RP	LF	HF	LF/HF	r_f_	β
Control subjects	0.09±0.01	0.13±0.05	0.24±0.09	0.12±0.06	2.3±0.8	0.3±0.1	0.4±0.1
Recently- diagnosed DM patients	0.08±0.01	0.06±0.03	0.15±0.06	0.07±0.04	2.6±0.9	0.17±0.07	0.26±0.08
Long-standing DM patients	0.06±0.01	0.01±0.02	0.09±0.06	0.04±0.03	2.2±0.9	0.09±0.06	0.08±0.06

The parametric Kolmogorov-Smirnov test (*z*,1.0) for a normal distribution was used to compare the study groups and to test the correlations related to the evolution DM. Student’s *t* test was used to compare the statistical measures of the IBI records from control subjects, recently diagnosed, and long-standing DM patients. A value of *p*<0.05 was considered to be statistically significant. Graphs and calculations of statistical parameters were realized in OriginPro^®^ 9.1, 64 bits, and were double checked with an independent Python^®^ program.

## Results

Table 1 resumes the frequency-domain parameters obtained during the controlled breathing test for the three subject groups. In the table, “Peak position” stands for the central frequency of the respiratory peak, as defined in the Methods section, “RP” indicates the area under the respiratory peak, “LF”, “HF” and “HF/LF” correspond to the traditional frequency-domain parameters, while r_F_ and β are our new proposed parameters. As can be glanced from the table, the position of the respiratory peak occurs around 0.1 Hz for the control group, as expected (since this is the breathing frequency), but gradually shifts towards lower frequencies as diabetes evolves, as observed also in Ref. [38]. Similarly, the area of the respiratory peak diminishes with the progress of the disease. This reflects the general trend of loss of spectral power also seen in the decrease of the values of the LF and HF parameters.

**Fig 3:**
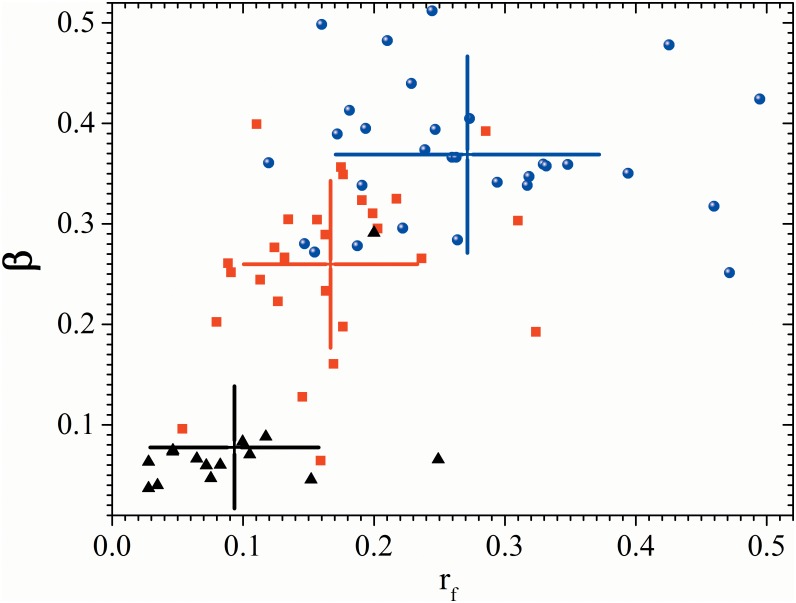
β parameter of all the patients. Scatter plot of β (breathing to total variability ratio) against r_f_ (frequency radius) during controlled breathing test for control subjects (blue dots), recently diagnosed DM patients (red squares) and long-standing DM patients (black triangles). Cross-hairs correspond to one standard deviation centered on the group average.

The traditional “HF/LF” ratio was not significantly altered in our tests: all three control groups exhibited values that are within the measurement errors of each other. On the other hand, our new parameters, r_f_ and β, both exhibit a statistically significant progressive decrease as the disease evolves (with p<0.05). The values of these two parameters thus characterize each of the test groups, specially permitting to distinguish between the control and advanced evolution test groups. This is further accentuated when they are used in combination. This is visually evident in Fig 3, where we have plotted the values of r_f_ and β for all subjects in two-dimensional space. Most of the individuals in each test group are clustered in distinct regions of the space, with long-evolution DM subjects clustered much closer to the origin than control subjects.

## Discussion

Recently, a meta-review was published that shows a gender difference in heart rate variability, in particular, women have a statistically significant lower total spectral power than men [39]. However, in the present study, differences between groups are established through the β parameter that quantifies for each subject the intensity of the peak in frequency domain associated with controlled breathing with respect to the total power spectral which might cancel out the gender effect. Since this parameter is defined as a ratio, it is not sensitive to the total spectral power of the series, and hence we do not expect these gender effects to be important for this measure. Our control and recently diagnosed study groups are relatively gender balanced, having 13 males vs. 17 females and 14 males vs. 16 females. For the long standing DM group, however, there is a gender bias (5 males vs. 10 females), but we suppose that the long-term effects of the disease will be more important than the gender difference. However, in future studies it would be of interest to analyze if there is a significant gender difference.

Control and recently diagnosed DM groups have the same average age (around 40 years). But the long-standing DM group has an age bias mainly because of the very strict inclusion and exclusion criteria that were applied, making it difficult to find elderly subjects that fulfill all the necessary requirements to be considered as control, and also because of the definition of the “long-standing” DM group (having being diagnosed at least 5 years prior to the study) which makes it harder to find young subjects for this group. From the few studies of short-term heart rate variability available, there is evidence that the scaling and nonlinear properties of the heart rate variability remain on average unchanged with respect to age [40]. However, other authors [41, 42] have shown that there is a statistical significant correlation with age of the traditional spectral indices (with LF+HF total power decreasing substantially with age). Since our parameter β essentially measures cardiorespiratory coupling there is no a priori evidence that it should be correlated with age. Moreover, the primary effect of age, i.e., diminishing the total power spectral density, also decreases the power in the respiratory peak in an approximately proportional way, so its ratio, as the β parameter, should be more influenced by disease and not by age.

We found no statistically significant differences between control subjects and diabetic patients for the supine rest and standing upright tests for none of the statistical parameters discussed above (student’s t test gives p>0.1); we thus focused our analysis in the results of the controlled breathing test. For this test, the traditional LF, HF and LF/HF measures are similar for all subjects.

The most striking difference we found in the Fourier spectra of the test subjects lies in the presence and strength of the cardiorespiratory coupling seen during the test. For the control subjects, a strong peak associated with the controlled breathing is present at the breathing frequency (this respiratory peak is not present in clinostatism and orthostatism records, since spontaneous normal respiration occurs faster than the imposed rhythmic breathing frequency of 0.1Hz). This peak gradually loses power as DM evolves over time, as can be appreciated by comparing the RP of recently diagnosed and long-standing DM II patients (see Table 1 and Fig 2). As diabetes evolves over time, the position of the respiratory peak is also shifted to frequencies smaller than 0.1 Hz (Table 1). This indicates that the modulation imposed by controlled breathing on the heart rate slows down in diabetic patients when compared to the control subjects. A similar result was found in diabetic patients with advanced neuropathy, which suggest that this may be due to the loss of conductive nerves or to changes in the conduction velocity [38]. Our proposed β parameter, designed to quantify the strength of the respiratory coupling, clearly indicates this correlation. When combined with the r_f_ parameter, designed to quantify the loss of power across the spectrum in a nonlinear fashion, spectral analysis of the respiration peak under the controlled breathing test offers a new statistically significant distinction between healthy and diabetic subjects (with p<0.05), as is visually evident in the scatter plot of Fig 3. Lastly, we note that the changes in these parameters are not statistically significant in the IBI records during the supine and standings tests, as expected from the observed lack (or weakness) of respiratory coupling during normal breathing. This strengthens the value of the controlled breathing test in identifying the changes caused by the evolution of DM.

## Conclusions

Breathing modulations at 0.1 Hz have been associated with an oscillation of the baroreceptor reflex. From the displacement of the RP peak in the diabetic patients, we deduce that the sympathetic activity that is driven by the baroreceptor may also be affected in DM patients. As diabetes evolves over time, the β parameter obtained during the controlled breathing test decreases, indicating a loss of the breathing modulation of the heart rate, and the r_f_ parameter diminishes, reflecting a reduction of the modulation capacity of the autonomous nervous system. Our analysis suggests that diabetes not only produces a more rigid heart rate with less variance, but, more importantly, also alters the coupling between breathing and the heart rate. These findings can be explained by a progressive decrease of vagal activity in the heart and possibly also by a gradual decrease of sympathetic activity given that the breathing frequency oscillation coincides with the baroreceptor activity. The new parameters r_f_ and the β are simple to evaluate and, in contrast to the traditional parameters (LF, HF, and LF/HF), allow to distinguish between control subjects, recently diagnosed and long-standing diabetic patients so they can be helpful for clinical diagnosis.

The results of this study indicate that the coupling of the respiratory and cardiac systems is significantly altered in diabetic patients, as shown mainly by the loss of the respiration peak in the power spectrum of IBI time series, and also by a reduction of the power of both the high-frequency (conventionally suggestive of cardiac parasympathetic) and low-frequency (conventionally suggestive of cardiac sympathetic) regions of the IBI spectrum. Given that a reduction in heart rate variability indices has been linked to increased all-cause and cardiac mortality, it is tempting to suggest that patients with diabetes who have a low HRV are at an increased risk of mortality.
